# Assessment on facile Diels–Alder approach of α-pyrone and terpenoquinone for the expedient synthesis of various natural scaffolds

**DOI:** 10.1007/s13659-022-00333-4

**Published:** 2022-03-31

**Authors:** Aluru Rammohan, Albert F. Khasanov, Dmitry S. Kopchuk, Duvvuru Gunasekar, Grigory V. Zyryanov, Oleg N. Chupakhin

**Affiliations:** 1 Ural Federal University, 19 Mira St., Ekaterinburg, 620002 Russian Federation; 2 Natural Products Division, Department of Chemistry, Sri Venkateswara University, Tirupati, 517502 India; 3 Ural Division of the Russian Academy of Sciences, I. Ya. Postovsky Institute of Organic Synthesis, 22 S. Kovalevskoy St., Ekaterinburg, 620219 Russian Federation

**Keywords:** α-Pyrone, Diels–Alder reaction (DAR), Marine natural compounds, Terpenoquinone, Total synthesis

## Abstract

The development of highly facile synthetic procedures for the expedient synthesis of complex natural molecules is always in demand. As this aspect, the Diels–Alder reaction (DAR) has a versatile approach to the synthesis of complex natural compounds and highly regio-/stereoselcetive heterocyclic scaffolds. Additionally, α-pyrone and terpenoquinone are two versatile key intermediates that are prevalent in various bioactive natural compounds for instance, (±)-crinine, (±)-joubertinamine, (±)-pancratistatin, (−)-cyclozonarone, and 8-ephipuupehedione, etc. Hence, the current review summarizes the Diels–Alder reaction application of α-pyrone and terpenoquinone to the constructive synthesis of various natural products over the past two decades (2001–2021). Equally, it serves as a stencil for the invention and development of new synthetic strategies for high-complex molecular structured natural and heterocyclic molecules.

**Graphical Abstract:**

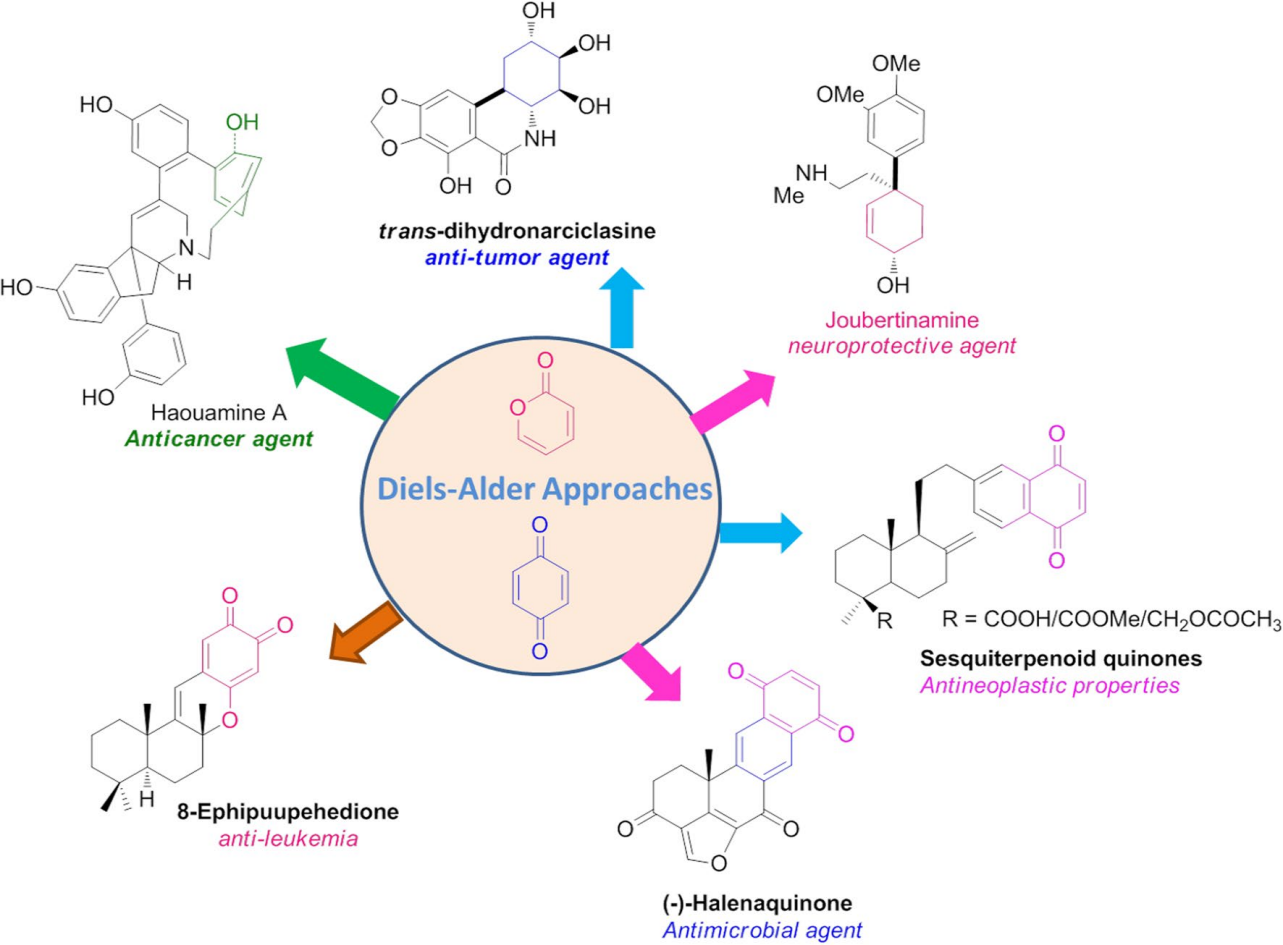

## Introduction

The development of innovative pharmaceutical agents from natural origin (like marine products) has played a tremendous role in the modern drug discovery. To date, a wide variety of complex marine natural products have been acknowledged as a lead agents to ameliorate the triggers of various disease like diabetes, microbial infections, cardiovascular disease, hypertension, immune related problems and neurological disorders, etc. [1, 2]. In this regard, α-pyrone (*syn.* 2-pyrones) and terpenoquinone compromising marine compounds have received considerable attention in the medicinal chemistry. Since, they have exhibited wide-variety of pharmacological activities such as antibiotic, anticancer, antimicrobial, antimalarial, and neuroprotective tactics [3, 4]. In addition, the analogues of α-pyrone and terpenoquinones have been accredited as an imperative bioactive-synthons in numerous complex natural products [5]. Therefore, the design and development of α-pyrone and terpenoquinone analogues have become an important strategy in current drug innovations through adaptive synthetic approaches [3, 6]. In this scenario, the Diels–Alder reaction is the most profitable approach for the facile synthesis of complex natural compounds with a pharmaceutical grade [7–9]. Furthermore, the DAR envisioned a highly-atom economical and creative transformation for the development of stereoselective novel drug agents [8, 9]. Likewise, the Diels–Alder reaction also has a wide choice of variety of industrial applications which includes hetero-DARs, intramolecular [4 + 2]^π^ cycloadditions, and catalytic reactions for the stereoselctive transformations. Thus, the Diels–Alder cyclization has an amazing strategy in synthetic organic chemistry and medicinal chemistry applications.

 Further, our efforts have continued towards in the Diels–Alder reactions [10–12], cycloadditions [13–15], and adeptness in the structural studies of bioactive natural products [16–20]. Therefore, the present appraisal aims to emphasize the role of Diels–Alder approach of α-pyrones and terpenoquinone in the constructive cycles of natural complexes. Equally, it highlights various Diels–Alder approaches for the design and development for bioactive natural compounds through medicinal chemistry approaches.

## Diels–Alder approach of α-pyrone to the pragmatic synthesis of natural compounds

**Fig. 1:**
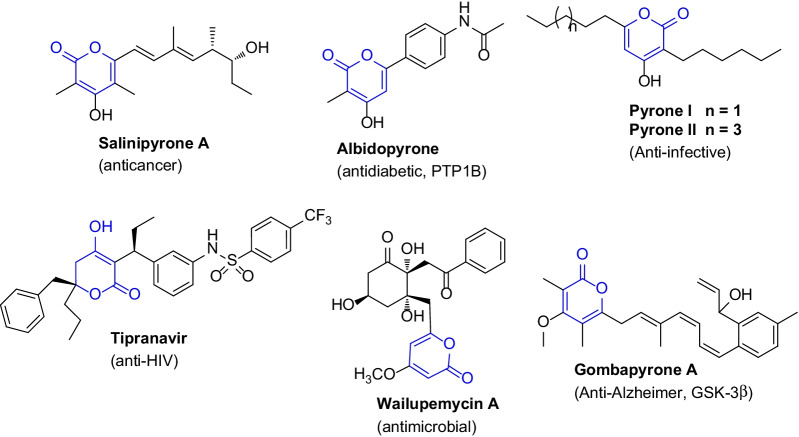
Structures of some nominated biologically potent α-pyrone marine compounds [3, 21]

The chromophore α-pyrone serves as a versatile building block in numerous bioactive natural marine products such as albidopyrone (antidiabetic), salinipyrone A (anticancer), wailupemycin A (antimicrobial), tipranavir (anti-HIV), pyrenes I–II (anti-infective), and gombapyrone A (glycogen synthase kinase-3*β* inhibitor) (Fig. 1) [3, 21]. Therefore, there is considerable interest among researchers in drug innovation owing to the unique structural and pharmaceutical properties of α-pyrone marine compounds. In addition, the developments of highly efficient synthetic tactics are needed to access the versatile analogues of bioactive α-pyrones. Considering all these prominence, an assessment of Diels–Alder approach for the expedient synthesis of α-pyrones are summarized as underneath.

**Scheme 1:**
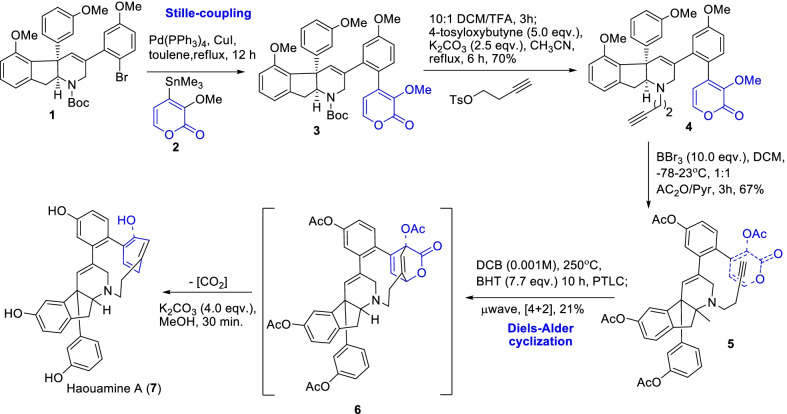
Synthesis of haoumine A involving pyrone-alkyne Diels–Alder cyclization

Baran and Burns demonstrated the constructive total synthesis of an important anti-cancer indeno-tetrahydropyridine analogue i.e., (±)-haouamine A (**7**) through a sequential reactions of Stille coupling of pyrone and Diel-Alder cyclization (Scheme 1) [22]. The introduction of α-pyrone chore **2** into the indeno-tetrahydropyrdine intermediate **1** by the Still coupling procedure was an important strategy in the synthesis of haouamine A. As well, another synthetic challenge was the unusual macrocyclization achieved through the pyrone-alkyne Diels–Alder reaction of **5**, which embedded leaving of CO_2_ group by a pseudo-boat configuration **6** and subsequent aromatization of viable precursor to **7**. Therefore, conferring to the biosynthetic origin the role of α-pyrone synthon was essential for the unusual oxygen pattern of highly strained macrocylic analogue **7** presence.

**Scheme 2:**
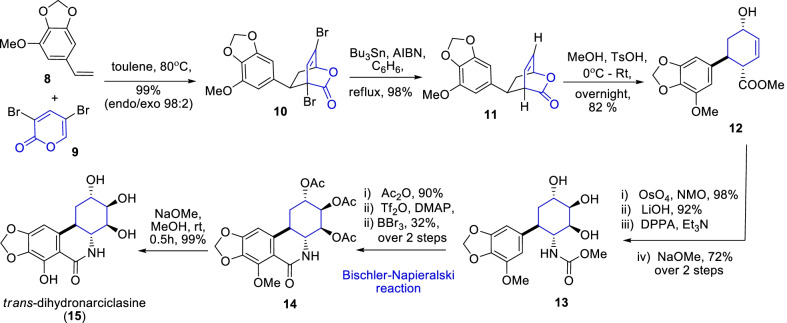
Synthesis of *trans*-dihydronarciclasine through *endo*-selective Diels–Alder cyclization

Equally, Shin and co-workers reported a total synthesis of the anti-tumor agent, *trans*-Dihydronarciclasine **15** over a Diel-Alder cyclization (Scheme 2) [23]. An important strategy in the synthesis of phenanthridone **15** was the outline of ring B accomplished through a high selective *endo*-adduct **10** in 99% yield by the Diels–Alder cyclization of α-pyrone derivative **9** with styrene derivative **8**. Further, the α,β-unsaturated cyclic adduct **10** was transformed into a methyl carbamate **13**, and then ensuing Bischler-Napieralski reaction of it acylated derivative **13** resulted the targeted *trans*-phenanthridone **15**. Later, Cho and his co-worker developed a more efficient route for large-scale production of **15** by enforcing the limitations of Bischler-Napisrealski cyclization reaction of the ester intermediate [24]. Therefore, from the total synthesis of **15**, it has been expanded that α-pyrone synthon **9** plays an essential role in the biogenesis of *trans*-dihydronarciclasine.

**Scheme 3:**
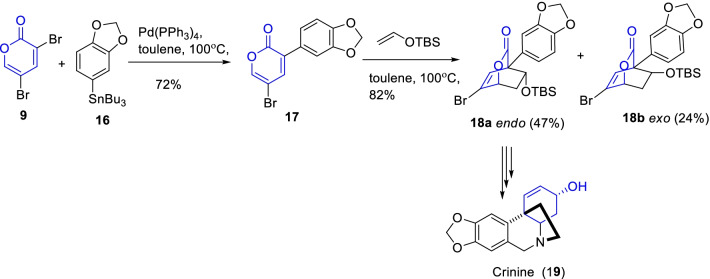
Synthesis of *endo*-bicyclolactone, a key intermediate of crinine by Still coupling and Diels–Alder cyclization methodologies

Further, Tam and Cho demonstrated another interesting natural antitumor alkaloid i.e., (±)-crinine (**19**) by Still coupling and Diels–Alder cyclization approaches (Scheme 3) [25]. Primarily, the synthesis of alkaloid **19** involves the regioselective coupling of the α-pyrone analogue **9** and aryltin derivative **16** prompted to the required α-pyrone diene **17** in 72% yield. Subsequently, the Diels–Alder cyclization of **17** with TBS vinyl ether occasioned the mixture of endo/exo-bicyclolactones (**18a**/**b**) in a 2:1 ratio. Further, the sequential reactions of *endo*-bicyclolactone **18a** provide the total synthesis of tetrahydroisoquinoline alkaloid **19**. Thus, from the stated synthetic approach, the regioselective pyrone-aryltin coupling and Diels–Alder cyclization plays a title role in the synthesis of *endo*-bicyclolactone **18a**, a key intermediate of (±)-crinine.

**Scheme 4:**
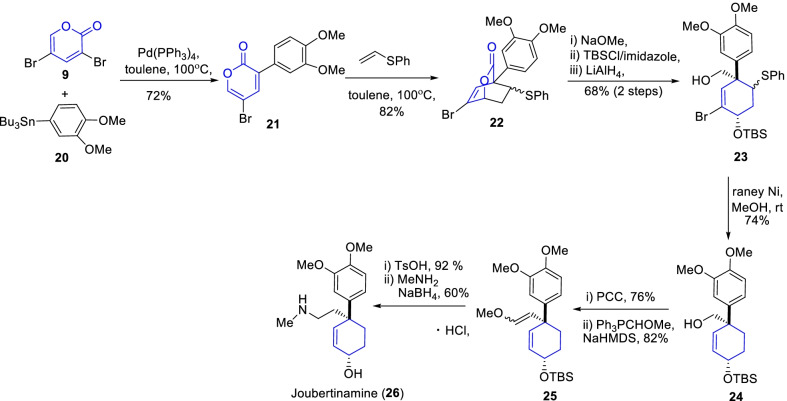
Synthesis of joubertinamine through Still coupling and Diels–Alder cyclization paths

Likewise, an sceletium alkaloid (±)-joubertinamine (**26**) has been accredited an pharmaceutically important agent to treat psychological disorders, anxiety, depressive state, alcohol and drug addictive conditions, and neurological disorders [26, 27]. Further, Tam and Cho deliberated the facile total synthesis of joubertinamine (**26**) over a Still coupling and Diels–Alder cyclization strategies (Scheme 4) [26]. As similar to the crinine (**19**) synthesis, the regioselctive coupling and Diels–Alder cyclization of α-pyrone **9** was facilitated the essential key cyclohexene intermediate **24**. Subsequently, the PCC oxidation and then witting reactions accomplished the target compound, joubertinamine **26**.

**Scheme 5:**
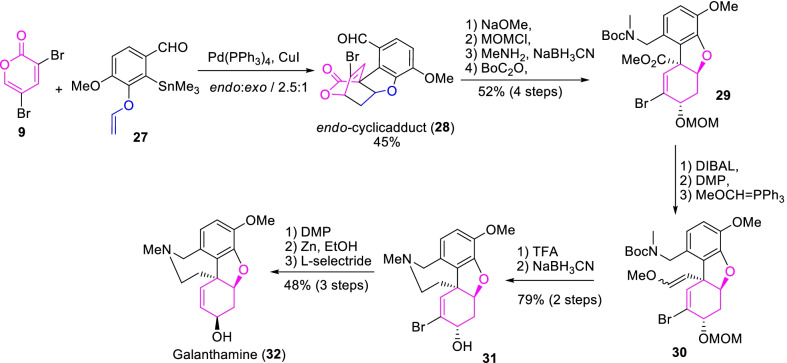
An efficient approach for the total synthesis of Galanthamine through tandem C3-selective Still coupling and IMDA approaches

Galanthamine is a biologically important cyclic tertiary amine class alkaloid used to treat the symptoms of Alzheimer disease [28]. In this regard, Chang et al.[29] demonstrated an efficient synthetic strategy for the total synthesis of galanthamine (**32**) through tandem C3-selective Still coupling and IMDA approaches as described in Scheme 5. Essentially, the *endo*-tetracyclolactone adduct **28** was achieved over a Stille coupling of α-pyrone **9** with aryl stannane **27**. Further, the ring-opening of a selective diastereomeric adduct **28** and then, followed by hydroxyl protection, amination and carbamate erection occasioned the respective, MOM ether and ester functionalized compound **29**. Then after, DIBAL reduction, Dess-Martin peroxidation (DMP) followed by Witting olefination caused in a diastereomeric mixture of enol ether derivative **30** in 46% yield. Similarly, accompanying TFA hydrolysis, reductive amination provided the tetracycle-alkaloid derivative **31**. Finally, the sequence reactions of DMP, debromination and the *L*-selectride reduction furnished galanthamine (**32**) in 48% yield. Therefore, the stereoselective tandem Still coupling/IMDA reaction of α-pyrone **9** was the key strategies to attain the *endo*-cyclic adduct **28** in the effective total synthesis of galanthamine.

**Scheme 6:**
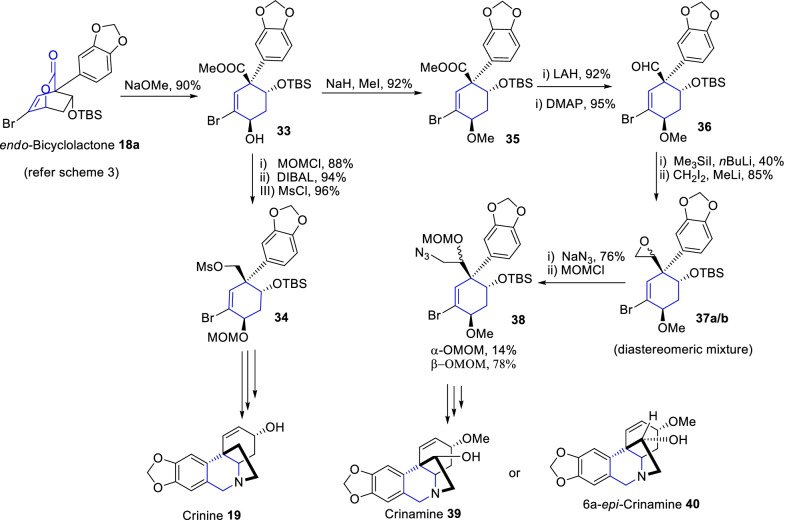
Synthesis of crinine-type alkaloids through an important enophile α-pyrone synthon derived Diels–Alder approach

Likewise, the continuing efforts of Tam and colleagues [30] have pronounced a unified approach to the total synthesis of various tetrahydroisoquinoline alkaloids such as (±)-crinine **19**, (±)-crinamine **39**, and (±)-6a-*epi*-crinamine **40** (Scheme 6). Primarily, the key bicyclolactone intermediate **18a** was achieved by Still coupling and Diels–Alder reaction of α-pyrone synthon **9** as described in Scheme 3. Further, the *endo*-bicyclolactone **18a** was transformed into respective key cyclohexene derivatives **33–38** as illustrated in scheme 6. Further, diverse sequential reactions were transformed into respective, crinine-type alkaloids **19**, **39** and **40**. Therefore, α-pyrone analogue was an imperative enophile synthon in the biogenetic Diels–Alder approach of various complex natural compounds.

**Scheme 7:**
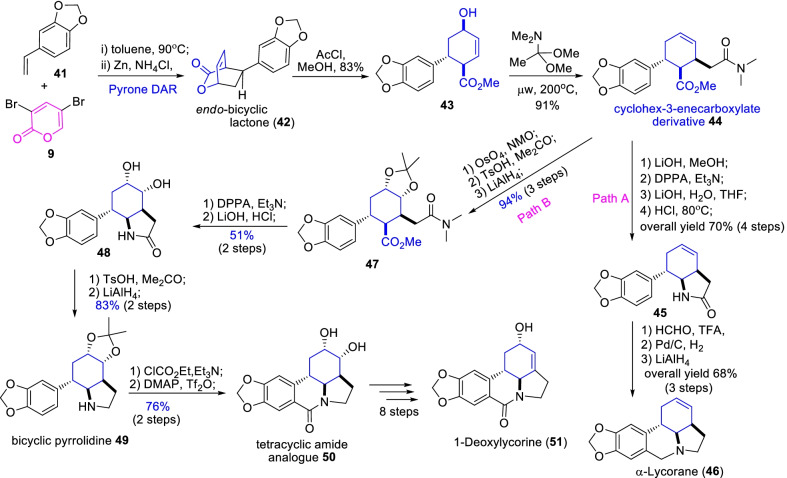
The efficient α-pyrone Diels–Alder approach for expedient synthesis of pyrrolo[de]phenanthridine alkaloids, α-lycorane and 1-deoxylycorine

Lycorine, lycorane, and 1-deoxylycorine are the most attention-grabbing and pharmacologically important pyrrolo[de]phenanthridine natural alkaloids [31, 32]. The total synthesis of α-lycorane (**46**) initiated by the Diels–Alder reaction of the α-pyrone derivative **9** with a styrene dienophile **41** which motivated the 10:1 mixture of diastereomeric cyclic adducts [32]. Further, the reductive debromination of nominated *endo*-cyclic adduct with Zn occasioned the desired bicyclic lactone **42**. Subsequent, acid-catalyzed methylation and the Eschenmoser–Claisen rearrangement prompted the important cyclohex-3-enecarboxylate derivative **44**. Consequent sequential reactions of Curtius rearrangement, lithium hydroxide treatment resulted in a bicyclic amide **45** as described in path A, Scheme 7. Further the amide **45** was imperiled to Pictet–Spengler reaction; Pd/C hydrogenation and LiAlH_4_ reduction accomplish the total synthesis of α-lycorane (**46**).

Equally, the key intermediate cycohex-3-enecrboxylate **44** was subjected to dihydroxylation with OsO_4_/NMO and the Curtius rearrangement motivated the diol lactam **48** in 51% yield [32]. Further, the protection of hydroxyl groups with TsOH/Me_2_CO and then, followed by carbonyl reduction with LiAlH_4_ led to the bicyclic pyrrolidine **49** as shown in path B, Scheme 7. The concomitant Bischeler–Napieralski reaction of bicyclic pyrrolidine **49** cyclized to tetracyclic amide analogue **50** in 76% yield. Finally, the amide derivative was subjected to a series of various 8 step-reactions such as protection; deprotection of hydroxyl, and reduction conditions were furnished the target derivative 1-deoxylycorine (**51**).

**Scheme 8:**
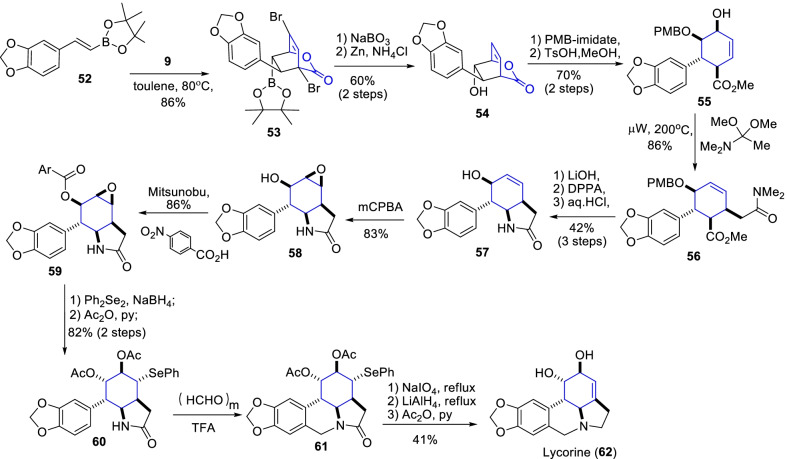
Synthesis of ( ±)-lycorine involving α-pyrone –Diels–Alder cyclization approach

Likewise, Shin et al.[33] demonstrated the amended total synthesis of ( ±)-lycorine (**62**) with the provision of chiral bicyclolactone alcohol **54** through Diels–Alder cyclization of pyrone **9** and *β*-borylstyrene **52** (Scheme 8). Further, the hydroxyl lactone **54** was subjected to acidic methanolysis and followed by Eschenmoser-Claisen rearrangement occasioned the key intermediate cyclohex-3-enecarboxylate derivative **56**. Subsequently, a sequence of reactions such as mCPBA epoxidation, Mitsunobu reaction, epoxide ring-opening, and Pictet-Spengler conditions afforded the tetracyclic lactam **61** in 70% yield. Finally, the LiAlH_4_ reduction of diacetate tetracyclic lactam **61** prompted the ( ±)-lycorine (**62**) at a yield of 41%.

**Scheme 9:**
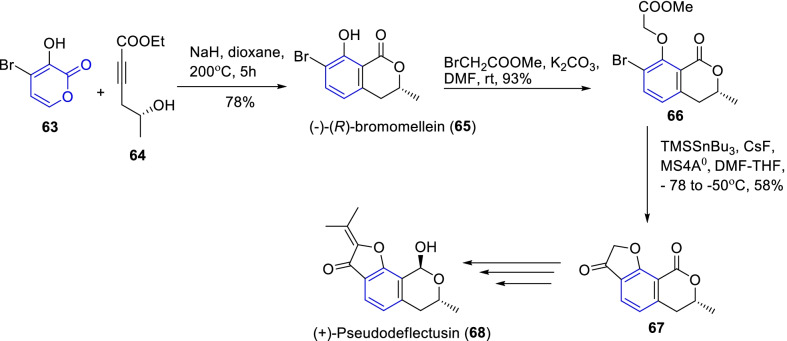
Synthesis of (+)-pseudodeflectusin through cascade reactions of regioselective DAR and lactonization accomplished from 7-hydroxy-α-pyrone

Sato and co-workers [34] demonstrated the total synthesis of another important anti-tumor scaffold (+)-pseudodeflectusin (**68**) by Diels–Alder and lactonization methods (Scheme 9). Primarily, the base-promoted Diels–Alder cyclization of 7-hydroxy-α-pyrone analogue **63** with an alkyne **64**, prompted the desired (-)-(R)-bromomellein **65** as an exclusively cyclic adduct in 78% yield. Further, the isochromanone adduct was adapted into tricyclic furanone intermediate **67** through the sequential reactions of alkylation with methyl bromoacetate, lactonization with TMSSnBu_3_/CsF in diffident conditions. Therefore, cascade reactions of regioselective DAR and lactonization accomplished from 7-hydroxy-α-pyrone (**63**) are prominent in the synthesis of ( +)-pseudodeflectusin **68**.

**Scheme 10:**
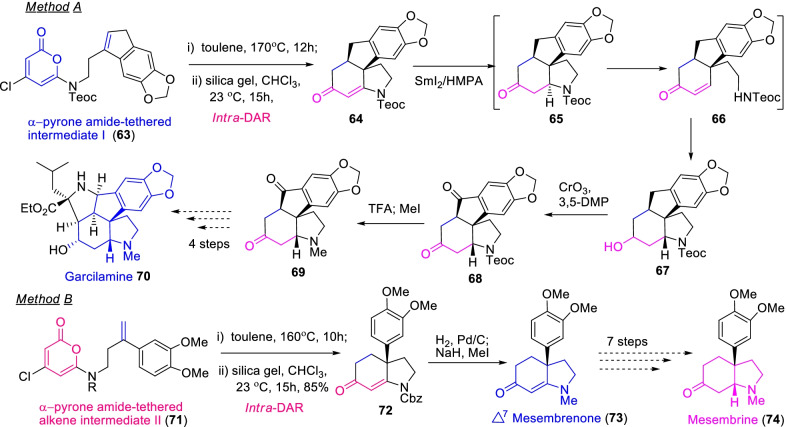
An efficient and expedient intramolecular α-pyrone-Diels–Alder cyclization approach for the synthesis of *Amaryllidacceae* alkaloids

Likewise, Gan et al. [35], established an efficient and expedient intramolecular pyrone Diels–Alder cyclization approach for the synthesis of *Amaryllidacceae* alkaloids viz*.*, garcilamine (**70**), Δ^7^-mesembrenone (**73**) and mesembrine (**74**) as described in Scheme 10. The adeptness and regioselectivity of the [4 + 2] cyclization depends on the substrate α-pyrone amide-tethered intermediate I (**63**) and II (**71**), which are readily accessible through augmented studies. Further, the sequential reactions of the Diels–Alder cyclic adduct (*i.e.* indole derivatives) were renovated to corresponding derivatives such as garcilamine, mesembrine and Δ^7^-mesembrenone. The success of the stated intramolecular Diels–Alder cyclization of α-pyrone analogues **63** and **71** have yielded diverse indole and hydroindole group alkaloids in a low step-count methodology.

**Scheme 11:**
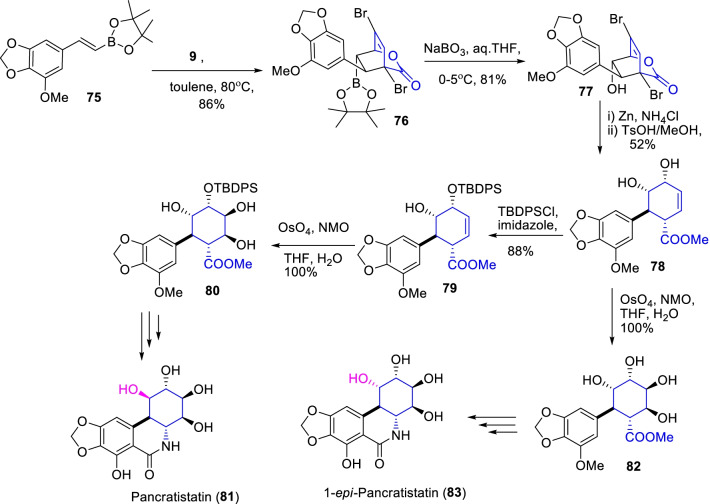
Synthesis of (±)-pancratistatin and (±)-1-*epi*-pancratistatin form Diels–Alder cyclization of *β*- borylstyrene with α-pyrone

Likewise, (±)-pancratistatin (**81)** and ( ±)-1-*epi*-pancratistatin (**83)** are two important anti-cancer *Amaryllidaceae* tricyclic alkaloids of natural origin [36]. Initially, Jung and co-workers [37] demonstrated the total synthesis of (±)-pancratistatin (**81**) by the cascade reactions of Diels–Alder cyclization, Curtius rearrangement and Bischler-Napieralski procedures. Later, the Cho group developed an advance synthetic procedure for both the (±)-pancratistatin (**81**) and (±)-1-*epi*-pancratistatin (**83)**, by identical reaction procedure with same starting materials of *β*-borylstyrene **75** (Scheme 11) [38]. Primarily, the dienophile *β*-borylstyrene undergoes DAR cyclization with α-pyrone (**9**, as diene) occasioned the *endo*-bicyclolactone **76** exclusively in 86% yield. Subsequent oxidation with sodium perborate stemmed the desired biclolactone alcohol **77** in 81% yield, and then the debromination, methanolysis primes to the key intermediate i.e., cyclohexene-diol **78**. Further, the Curtius rearrangement and Bischler-Napieralski reactions of corresponding tetraol intermediates occasioned the targeted alkaloids **81** and **83**, respectively. Therefore, the stated total synthesis of **81** and **83** became worthwhile with the formation of *endo*-cyclicadduct **76** in the inverse electron demand Diels–Alder cyclization of the α-pyrone derivative **9** with *β*-borylstyrene **75**.

**Scheme 12:**
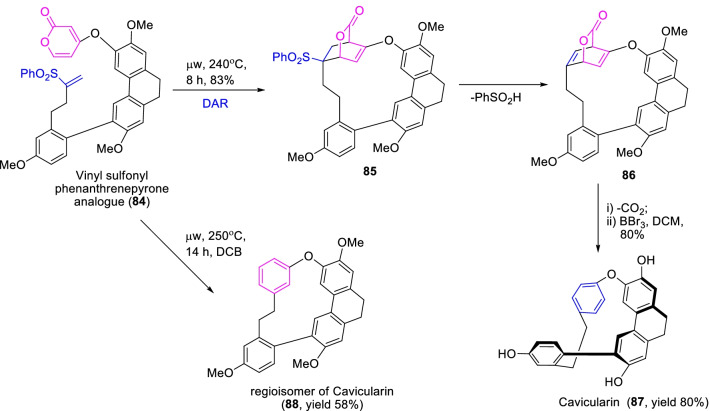
The microwave accustomed intramolecular Diels–Alder cyclization approach for expedient synthesis cavicularin

Further, conformationally chiral molecule cavicularin **87** has been reported to attract the attention of researchers due to its unique molecular architecture and interesting biological activities [39, 40]. As a result, Zhao and Beaudry [40], demonstrated a facile synthetic strategy for chiral macrocyclic bis(bibenzyl) derivative, cavicularin (**87**) by a controlled regiochemical approach of intramolecular Diels–Alder reaction as described in Scheme 12. Initially, the appropriate key Diels–Alder substrate of vinyl sulfonyl and α-pyrone substituted phenanthrene analogue **84** was achieved by a sequential reactions like Claisen-like condensation and Horner-Wadsworth-Emmons reaction procedures. Further, the intramolecular Diels–Alder cyclization of cascade substrate under microwave conditions occasioned the cavicularin **87** in 80% yield and its regioisomer **88** at a yield of 58%, respectively. Therefore, the pyrone Diels–Alder substrate **84** is essential for the construct of conformationally macrocyclic bis(bibenzyl) natural metabolites.

**Scheme 13:**
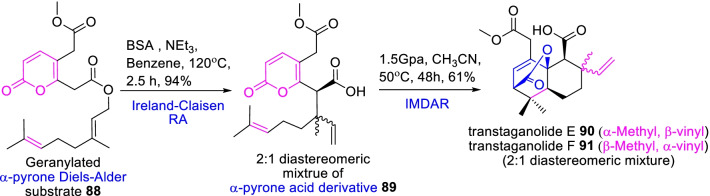
An IMDA cyclization of a geranylated α-pyrone Diels–Alder substrate for the facile synthesis of transtaganolides E and F

Basiliolide and transtaganolides are pharmacologically important natural metabolites with a novel framework of oxabicyclo[2.2.2]octene core derivatives [41]. Thus, the concise strategies and stoichiometric reagents are required to accomplish the total synthesis of unusual complex tricyclic substrates on an industrial scale. As this aspect, Larsson et al. [42] proposed a strategic synthesis for transtaganolides E (**90**) and F (**91**) that were potentially beneficial as analogue synthons for basiliolides and transtaganolides. Initially, a geranylated α-pyrone Diels–Alder substrate **88** was imperiled to Ireland–Claisen rearrangement to attain a rearranged α-pyrone acid derivative **89**. Further, the high pressure 1.5 GPa/50 °C conveys an IMDA cyclization accomplished the 2:1 diastereomeric mixture of transtaganolide E and F in 61% yield as illustrated in Scheme 13.

**Scheme 14:**
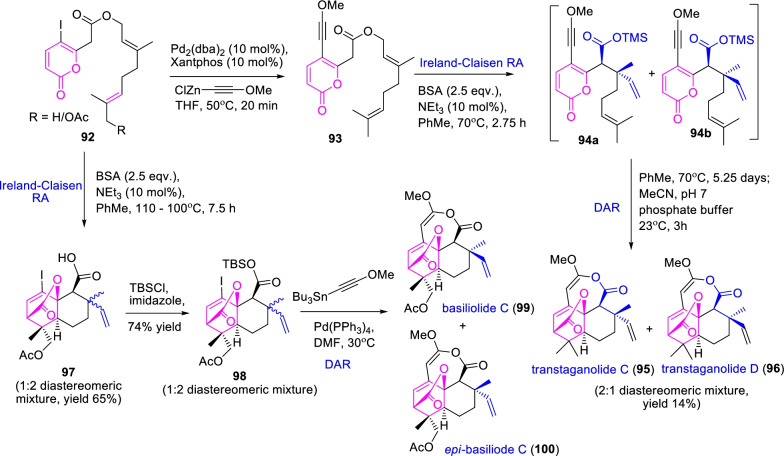
Total synthesis of transtaganolide and basiliolide class-compounds by Ireland-Claisen rearrangement (ICRA) and Diels–Alder cascade approaches

Further, Gordon et al. [43] shortened the total synthesis of transtaganolide and basiliolide class-compounds through Ireland-Claisen rearrangement (ICRA) and Diels–Alder cascade approaches as described in Scheme 14. Initially, the pyrone Diels–Alder substrate **92** with electron donating groups was achieved by Negishi cross-coupling, and the subsequent one-pot tandem ICRA and Diels–Alder sequence reactions resulted in 2:1 diastereomeric mixture of transtaganolides C (**95**) and D (**96**). Equally, the acrylated α-pyrone Diels–Alder substrate **92** under Ireland-Claisen condition provided a 1:2 mixture of C8 diastereomeric **97** in 65% yield. Further, the diastereomeric mixture was transformed into corresponding tricyclic silyl esters **98**, and then palladium driven [5 + 2] annulation caused the basiliolide C (**99**) and *epi*-basiliolide C (**100**), respectively. Thus, the α-pyrone Diels–Alder template **92** and its electron-donating methoxy alkynyl group play a key role in the facile synthesis of the structurally complex transtaganolides and basiliolodes.

**Scheme 15:**
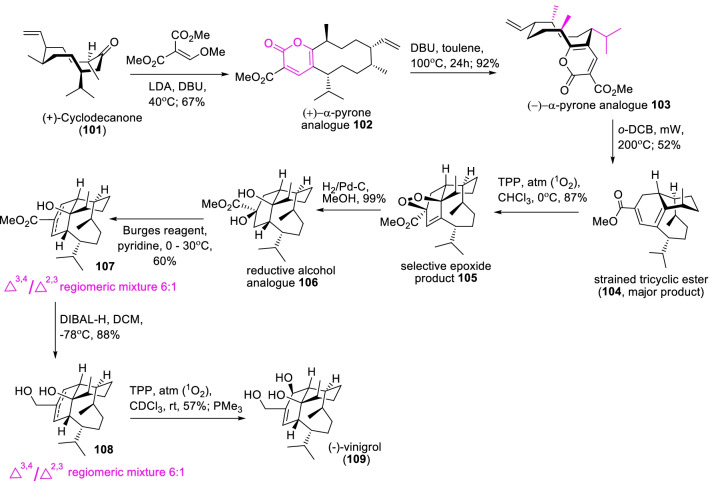
A facile transannular Diels–Alder cyclization route for the synthesis of (−)-vinigrol

Similarly, vinigrol (**109**) is another interesting natural molecule with a complex molecular framework and is prominent as a potent antihypertensive and antitumor agent [44]. To the expedient synthesis of continuous stereogenic tricyclic triterpenoid **109**, Xu et al. [45], proposed a facile transannular Diels–Alder cyclization procedure as illustrated in Scheme 15. Primarily, the key Diels–Alder template of α-pyrone analogue **102** was achieved over a Boger’s lactonization procedure of highly strained cyclodec-5-enone **101** with dimethyl methoxymethylenemalonate. The subsequent epimerization reaction of the (+)-α-pyrone analogue **102** in DBU/toluene at 100 °C occasioned the expected (−)-α-pyrone derivative **103**. Further, conducting the transannular Diels–Alder cyclisation of epimerized pyrone derivative **103** in DCB/mW at 200 °C procured the strained tricylic ester **104** as major product. Succeeding, selective epoxidation by ^1^O_2_, reductive cleave peroxide linkage, and directive Burgess’s reaction conditions are the sequence reactions concerning to the completion of the total synthesis of (−)-vinigrol (**109**). Therefore, the epimerized product (−)-α-pyrone analogue **103** synthesis and transannular Diels–Alder reaction are the key targets in the synthesis of highly strained tricyclic diterpenoid i.e. (−)-vinigrol.

**Scheme 16:**
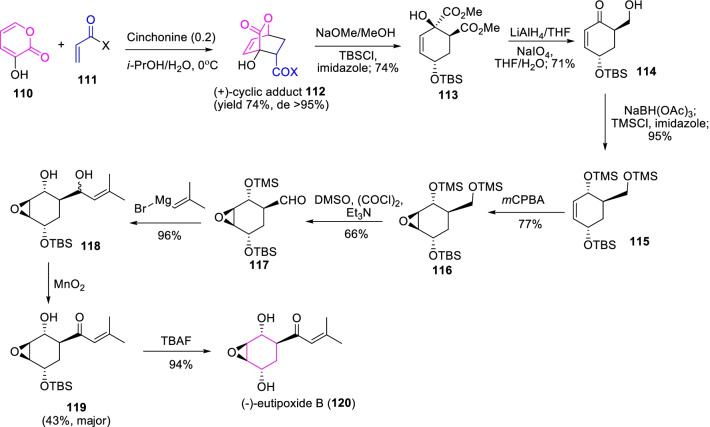
The total synthesis of eutipoxide B through the base promoted *asymmetric*-Diels–Alder cyclization of α-pyrone approach

Another interesting biologically active oxygenated cyclohexene epoxide, eutipoxide B (**120**) was widely produced by phytopathogenic fungus *Eutypa lata*[46]. Consistently, Shimizu et al.[47] projected the total synthesis of eutipoxide B (**120**) through the base cinchonine promoted *asymmetric*-Diels–Alder cyclization of 3-hydroxy-2-pyrone **110** with electron deficient dienophile **111** convinced the optically active cyclicadduct **112** at a yield of 74% (Scheme 16). Consequent reactions such as methylation, reduction of silyl ether derivative and oxidative cleavage, followed by epoxidation and Swern oxidation were prompted the chiral epoxy cyclohexane-3-carbaldehyde **117**. Further, treatment with 2-methylpropenyl Grignard reagent and deprotection of TBS ether resulted in a 94% yield of the desired (−)-eutipoxide B (**120**). Though, the base catalyst cinchonidine used rather than cinchonine, the Diels–Alder reaction results the (−)-cyclicadduct with 82% yield and > 95% diastereomeric excess, and the succeeding sequence reactions occasioned the (+)-eutipoxide B. Therefore, the efficient and regioselectivity of asymmetric Diels–Alder reaction of 3-hydroxy-2-pyrone with dienophile presents a key role in the synthesis of chiral oxygenated cyclohexene epoxide metabolites.

**Scheme 17:**
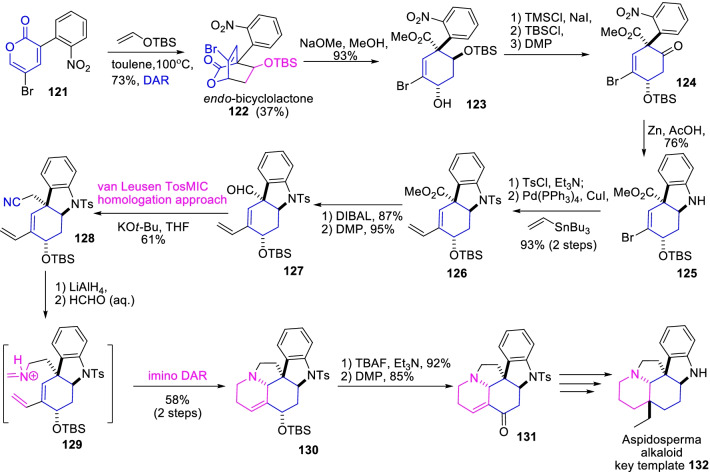
The *imino*-Diels–Alder cyclization impelled synthesis of pentacylic enone derivative, an analogue of *Aspidosperma* alkaloid

Similarly, Tam et al. [48] demonstrated an efficient strategic synthetic approach for the pentacyclic enone intermediate **131** towards biologically imperative *Aspidosperma* alkaloid **132** (Scheme 17). The synthesis was commenced with the attainment of *endo*-bicyclolactone **122** in 37% yield by the Diels–Alder cyclization of 3-(2-nitrophenyl)-5-bromo-α-pyrone **121** with silyl vinyl ether. Further, the chronological reactions counting methanolysis, hydroxyl protection, and the peroxide oxidation, and Zn-reduction were driven the indole ester derivative **125** construction. Subsequently, the Still coupling with vinyl stannate, ester-group reduction, and followed by van Leusen TosMIC homologation conditions were prompted the nitrile analogue **128** in 61% yield. Likewise, reduction of nitrile, and then heating with aqueous formaldehyde impinged the *imino*-Diels–Alder cyclization prompted the formation of important pentacylic enone derivative **131**. Therefore, the α-pyrone Diels–Alder cyclization plays a key role in pentacyclic enone intermediate synthesis for the proposed *Aspidosperma* alkaloid.

**Scheme 18:**
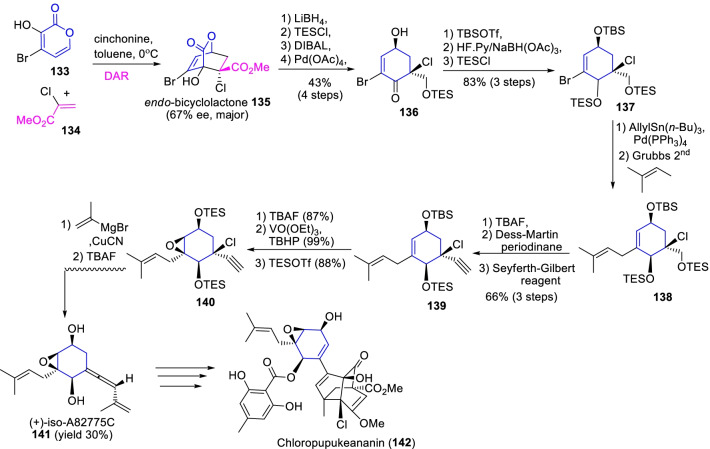
The intermolecular Diels–Alder reaction of α-pyrone assisted prominent strategies for the synthesis of (+)-iso-A82775C, a key intermediate of chloropupukeananin

Equally, (+)-iso-A82775C (**141**) is a fascinating diastereomic cyclohexene epoxide derivative, deliberated an important biosynthetic intermediate of various drugs for instance chloropupukeananin, pestaloficinols, and pestalofones, etc. [49]. Further, it displays an essential role in the biosynthesis of chloropupukeanin (**142**), a potent inhibitor of HIV-1 replication and human tumor cells pathogenesis [50]. Given the importance of fungal metabolite A82775C, Suzuki et al. [51] commenced its total synthesis through enantioselective Diels–Alder cyclization, Stille coupling and cross-metathesis approaches as described in Scheme 18. Principally, the Diels–Alder reaction of 4-bromo-3-hydroxy α-pyrone **133** with methyl 2-chloroacrylate **134** occasioned the optically active endo-cyclic adduct **135** at 67% ee with the presence of cinchonine base. Further, the sequential reactions such as TES protection, DIBAL reduction, Criegee oxidation by Pb(OAc)_4_ occasioned the cyclohexanone derivative **136** in 43% yield. Afterwards, the diastereoselective reduction of ketone derivative with NaBH(OAc)_3_, followed by TES protection of hydroxyls ensued the 1,3-diol **137**. Likewise, the Stille coupling with allylSn(*n*-Bu)_3_ and Cross-metathesis by Grubb’s catalyst (II) were prompted the prenylcyclohexene **138**. As well, the consecutive reactions like Dess-Martin oxidation, Seyferth-Gilbert homologation, and VO(OEt)_3_/TBHP epoxidation gave the exclusive diastereomer **140**. Finally, *anti*-selective copper facilitated S_N_2′ reaction of diastereomeric epoxide **140** and the TBAF deprotection reactions succeeded the (+)-iso-A82775C (**141**) synthesis in 30% yield. Therefore, the intermolecular Diels–Alder reaction of α-pyrone **133** and the sequential metalation reactions are the prominent strategies to achieve the (+)-iso-A82775C of cholropupukeananin (**142**) synthesis.

**Scheme 19:**
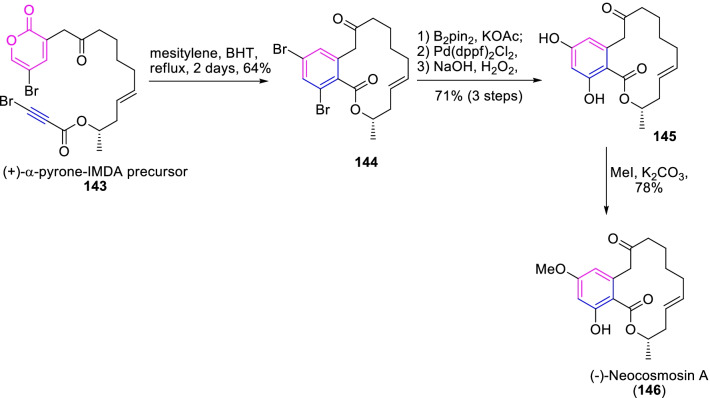
The IMDA cyclization α-pyrone approach to the expedient synthesis of (−)-neocosmosin A

As well, a resorcyclic acid lactone (−)-neocosmosin A (**146**) was isolated from the fungus Neocosmospora sp., and has been shown to have strong binding properties with cannabinoid receptors and human opioid [52]. As this aspect, Lee and Cho [53], demonstrated an efficient and rapid access to neocosmosin A through IMDA and cycloreversion approaches as described in Scheme 19. The target synthesis was motivated by the achievement of chiral-IMDA α-pyrone substrate **143** by various optimized studies. Consequently, the IMDA reaction of α-pyrone bromopropiolate substrate **143** gave the corresponding dibromobenzo macrocyclic lactone **144** in 64% yield. Further, on exposed to Miyaura reaction and then followed by oxidation of borate derivative prompted the (−)-macrocyclic resorcinol **145** in 71% yield. Finally, the perceptive methylation of less-hindered hydroxyl with MeI/K_2_CO_3_ accomplished the (−)-neocosmosin A (**146**) in 78% yield. Therefore, the intramolecular Diels–Alder reaction of the α-pyrone substrate to achieve the macrolides like neocosmosin A is an efficient synthetic strategy.

## Diels–Alder approach for the expedient terpenoquinone arbitrated natural compounds

**Fig. 2:**
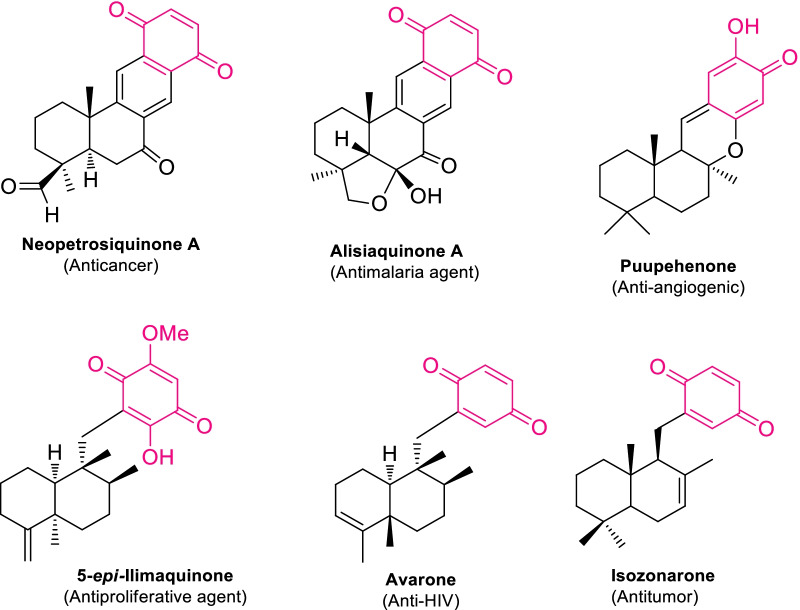
Some examples of terpenoquinone articulate bioactive natural molecules [6, 56–59]

As well, terpenoquinone is another interesting stencil found in numerous marine natural products like sesquiterpene benzoquinones, meroterpenes, merosesquiterpenes, norsesquiterpenes, and tetracarbocyclics, etc. [54–56]. Therefore, the substantial attention has been paid to the terpenoquinone cohesive natural compounds due to its extensive pharmacological properties [6, 56]. In this regard, various studies have revealed that certain marine sponges were richest source of bioactive terpenoquinones that imperative as antibacterial, anticancer, antitumor, antimalarial, and anti-HIV therapeutic agents [6, 56–59]. Therefore, some examples of isolated terpenoquinones and their pharmacologically significance are appended in Fig. 2. Considering the structural diversity and biological prominence of the natural terpenoquinone, the standing review emphasized the application of Diels–Alder cyclization approach to its expedient synthesis. In addition, the terpenoquinones are resourceful dienophiles that triggered lavish DAR approaches to the constructive complex natural products. Further, the Diels–Alder reaction was a facile synthetic approach for the quick generation of regio- and steroselective complex products with creditable yields.

**Scheme 20:**
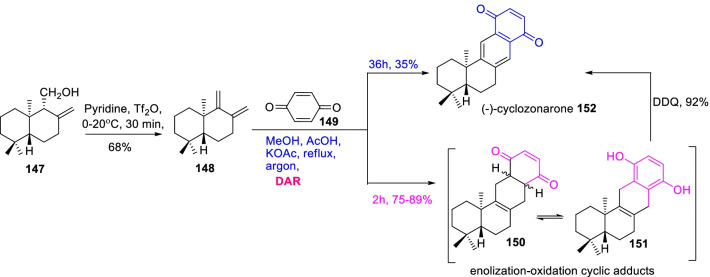
Diels–Alder reaction approach for the synthesis of (−)-cyclozonarone

From this aspect, a bioactive sesquiterpene quinone *i.e.* cyclozonarone (**152**) was widely distributed in marine algae *Dictyopteris undulata* [60], and it absolute configuration was (−)-(5*R*,10*R*)-cyclozonarone revealed by Cortes et al. [61] over an enantioselective synthesis. Later, Schroder et al. [62] demonstrated the fruitful total synthesis of (−)-cyclozonarone through an expedient Diels–Alder cyclization approach as illustrated in Scheme 20. Initially, the dehydration reaction of ( +)-albicanol **147** with Tf_2_O/pyridine occasioned the drima-(8,12), (9,11)-diene **148** in 68% yield, which then subjected to Diels–Alder reaction with benzoquinone **149** resulted a mixture of enolization-oxidation cyclic adducts **150** and **151** in 75–89% yield. Subsequently, on oxidation of cyclic adduct mixture with DDQ primes to (−)-cyclozonarone **152** in 92% yield. Whereas, the targeted sesquiterpene quinone **152** was achieved in 35% yield on extending the Diels–Alder reaction time to 36 h without subsequent DDQ oxidation. Therefore, the pragmatic synthesis of **152** was achieved through a controlled Diel-Alder cyclization of diene derivative with benzoquinone over a static reaction period as described in Scheme 20.

**Scheme 21:**
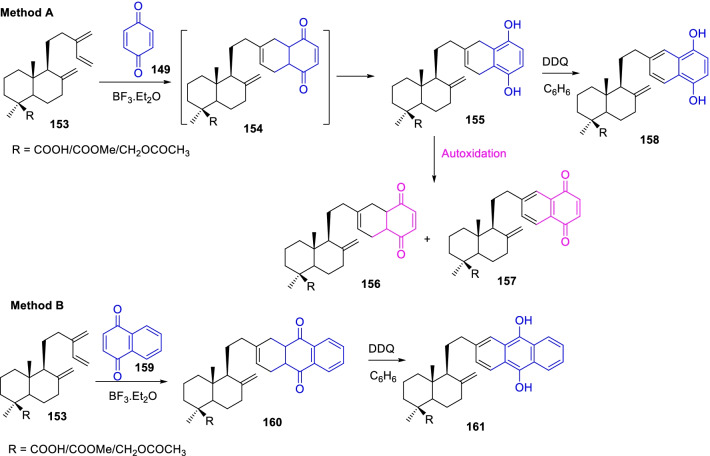
The facile Diels–Alder cycloaddition procedure for the synthesis of sesquiterpenoid quinones/hydroquinones

Likewise, Miguel del Corral et al. [63], demonstrated the facile Diels–Alder cycloaddition procedure for sesquiterpenoid quinones/hydroquinones with interesting antineoplastic properties (Scheme 21). Primarily, the cycloaddition reaction of three labdanic diterpenoids **153** with *p*-benzoquinone **149** occasioned the corresponding hydroquinones **155** together with autoxidized quinones **156** and **157** as described in method A, Scheme 21. Further, the oxidation of hydroquinones **155** with DDQ was stemmed to the respective naphthohydroquinone **158**. Also, the Diels–Alder reaction of myrceocommunic derivatives **153** with naphthoquinone **159** was stimulated the respective diterpenyl anthraquinone **160** and hydroxyanthraquinone **161** as illustrated in method B, Scheme 21. In addition, the stated diterpenylquinones (**156**–**158**) and diterpenylhydroquinones (**160** and **161**) have been found to be substantial cytotoxic in 0.1–21 µM against various human tumor cells such as lung carcinoma (A-549), colon carcinoma (HT-29), murine leukemia (P-388), and malignant melanoma (MEL-28).

**Scheme 22:**
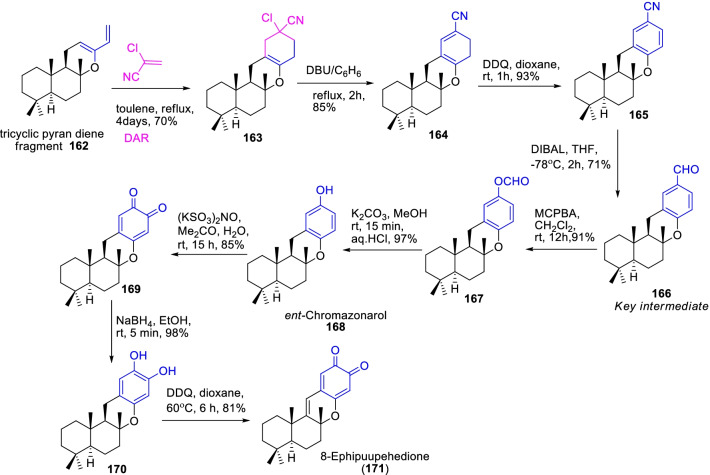
Diels–Alder cyclization procedure for the synthesis of an active aldehyde intermediate of 8-Ephipuupehedione

Likewise, another marine anti-leukemia sesquiterpene 8-ephipuupehedione **171** was found to be a potent inhibitor of cell-proliferation and associated cancer-pathogenesis paths [64]. As the aspect, Alvarez-Manzaneda et al.[65], demonstrated an facile Diels–Alder cyclization procedure for the synthesis of aldehyde intermediate **166**, an essential key synthon for the formation of marine metabolites like *ent*-chromazonarol **168** and 8-ephipuupehedione **171** as shown in Scheme 22. Primarily, the tricyclic pyran diene fragment **162** was synthetized from sclareol oxide, which then cycloaddition with α-chloroacrylonitile (dienophile) by DAR procedure provided the regioselctive cyclic adduct **163** in 70%. Afterwards, the successive treatments of cyclic adduct with DBU/C_6_H_6_, DDQ/dioxane and DIBAL/ THF stemmed the essential key aldehyde intermediate **166** in 71% yield. Therefore, the Diel-Alder cyclization was the static approach that ensued **166** in persuasive yields. Subsequent, Baeyer–Villiger oxidation of **166**, saponification, and DDQ oxidation were motivated the 8-ephipuupehedione metabolite **171**.

**Scheme 23:**
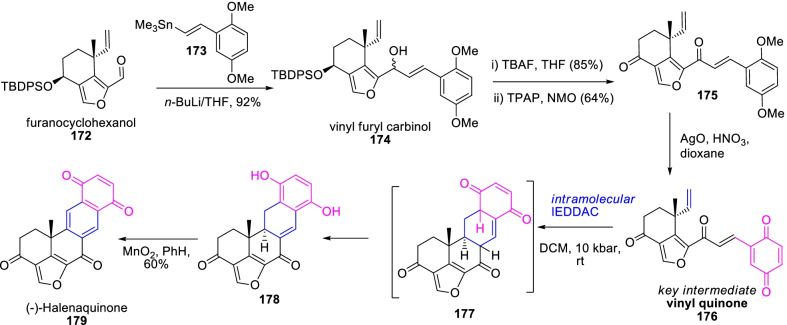
Diels–Alder reaction approach for the concise synthesis of (**−**)-halenaquinone

As well, the halenaquinone (**179**), a marine pentacyclic polyketide metabolite with unusual molecular structure, has been acknowledged as a potent antimicrobial agent [66]. Further, Kienzler et al. [67], demonstrated the asymmetric total synthesis of (−)-halenaquinone **179** through inverse-electron demand Diels–Alder cyclization (IEDDAC) approach as labelled in Scheme 23. Primarily, the vinyl furyl carbinol **174** was achieved in 92% yield through C–C functionalized organometallic coupling of pre-prepared [65, 68] furanocyclohexanol **172** and aryl vinyl stannane **173**. Succeeding desilylation, oxidative demethylation, and metal oxidation of secondary hydroxyls occasioned the highly stable key intermediate vinyl quinone of **176**. Auxiliary, the high-pressure 10 kbar driven intramolecular IEDDAC resulted in the respective tetracyclic adduct **178** at rt, and the subsequent oxidization with MnO_2_/PhH afford the aromatized (−)-halenaquinone (**179**) in 60% yield.

## Conclusions

In essence, the Diels–Alder reaction is a versatile synthetic approach to construct the highly complex molecular structures of bioactive natural compounds for clinical and therapeutic applications. Further, the existing assessment highlighted the role of α-pyrone and terpenoquinone in the synthesis of important bioactive natural compounds by Diels–Alder approach. Moreover, the present review may be beneficial as a template for the future development of new therapeutic leads, and as a key appliance for their drug discovery challenges.

## Abbreviations

AIBN: Azobisisobutyronitrile
BHT: Butylated Hydroxytoulene
DAR: Diels–Alder reaction
DBU: 1,8-Diazabicyclo[5.4.0]undec-7-ene
DIBAL: Diisobutylaluminium hydride
DMAP: 4-Dimethylaminopyridine
DPPA: Diphenylphosphoryl azide
HIV: Human Inmmunodeficiency Virus
IEDDAC: Inverse electron demand Diels–Alder cyclization
NaHMDS: Sodium bis(trimethylsilyl)amide
NMO: N-Methylmorpholine-N-oxide
TABF: Tetrabutylammonium fluoride
TBSCl: Tert-butyldimethylsilyl Chloride
TBDPSCl: Tert-butyldiphenylsilyl Chloride
TPAP: Tetrapropylammonium perruthenate
TMSSnBu_3_: Trimethylsilyltri-n-butyltin
